# Knowledge and practice of informed consent by physiotherapists and therapy assistants in KwaZulu-Natal Province, South Africa

**DOI:** 10.4102/sajp.v75i1.1330

**Published:** 2019-08-12

**Authors:** Kayode S. Aderibigbe, Sylvester C. Chima

**Affiliations:** 1 School of Nursing and Public Health, College of Health Sciences, University of KwaZulu-Natal, Durban, South Africa; 2 King Edward ѴІΙІ Hospital, Department of Physiotherapy, KwaZulu-Natal Department of Health, Durban, South Africa; 3 Nelson R Mandela School of Medicine, College of Health Sciences, University of KwaZulu-Natal, Durban, South Africa

**Keywords:** physiotherapy, informed consent, knowledge, clinical practice, public health, South Africa

## Abstract

**Background:**

Informed consent is a doctrinal prerequisite in accordance with the *National Health Act* 2003 and professional ethical guidelines. Current regulations stipulate that healthcare professionals obtain informed consent from patients prior to treatment. Misconduct charges relating to inadequate information disclosure have been recorded against South African physiotherapists.

**Objectives:**

This study evaluated knowledge and barriers to informed consent practice among physiotherapists and assistants at Ethekwini District public health institutions.

**Methods:**

This cross-sectional study utilised self-administered questionnaires. Statistical Package for Social Sciences was used to analyse variables. Significance level and attitude correlation was determined using chi-squared tests, Pearson’s correlation and Spearman’s coefficient.

**Results:**

Forty-nine respondents (43 physiotherapists, 3 technicians, 3 assistants) completed this study. Mean age and professional experience of respondents were 38 and 14 years, respectively. The majority were female (93%); 56% spent 5 to 10 min obtaining informed consent, mostly verbally (89%); while 47% correctly identified age of consent to routine treatment (12 years). Information provided to patients by respondents included treatment benefits (100%), common risks (81%) and ‘all material risks’ (31%). Fifty per cent of respondents showed positive attitudes to informed consent.

**Conclusions:**

Some practising physiotherapists and assistants in KwaZulu-Natal public healthcare institutions had only partial knowledge of informed consent regulations and local laws. Barriers to informed consent included language and excessive workload.

**Clinical implications:**

Patient-centred care is quality healthcare, and adequate informed consent knowledge improves clinical outcomes, respects patients’ dignity and autonomy. Continued professional education on healthcare law and ethics should be provided to practising physiotherapists and assistants.

## Introduction

Healthcare practice without informed consent (IC) may be an indication of a violation of basic human rights, legal requirements and ethical practice (Fremgen 2012). Such violations cannot, however, be isolated from the daily challenges encountered by all healthcare professionals (HCPs), such as time constraints, workload, cultural barriers and language difficulty, among others (Chima 2013). The Healthcare Professions Council of South Africa (HPCSA) guidelines (2008) require that IC must be obtained except in cases where this requirement has been waived. Similarly, the *National Health Act* (2003) (hereinafter NHA) stipulates that: ‘Health service may not be provided to a user without the user’s informed consent’, and that:

[*E*]very health care provider must inform a user of the user’s health status except in circumstances where there is substantial evidence that the disclosure of the user’s health status would be contrary to the best interests of the user. (NHA 2003:s.6)

Few empirical studies have documented the practice of IC among medical doctors, nurses and patients in South Africa (Chima 2013, 2015, 2017; Henley et al. 1995). However, investigations around IC among physiotherapists (PTs) have largely focused on qualitative studies (Barnitt & Partridge 1997; Delany 2005).

Some South African PTs have been charged with misconduct related to IC as reported by Hoffmann and Nortjé (2015). These charges related to treatment without full information disclosure and the patient’s permission, and were considered a violation of patients’ rights to doctrinal due process. In addition, it is a demonstration of a breach of the ethical principle of respect for autonomy (Chima 2009; Fremgen 2012).

Beauchamp and Childress (2009) have suggested that while information disclosure primarily represents the legal context of IC, in the moral viewpoint, it is about choices based on autonomy of healthcare users and not choices based on liability. Violation of individual autonomy of patients and violation of their bodily integrity on presentation before a court of law may be adjudicated as assault or battery (Fremgen 2012; Park et al. 2016). Various studies on targeted groups of HCPs, such as medical doctors and nurses, on knowledge of IC have concluded that there seems to be some deficiency of knowledge among HCPs in South Africa (Chima 2013, 2018a; Henley et al. 1995), as well as in other jurisdictions. This is as demonstrated by the recent judgment by the UK Supreme Court (Scotland), which held in the case of *Montgomery v Lanarkshire* (2015), that decisions pertaining to medical doctors with regard to IC practice are equally applicable, *mutatis mutandis*, to all HCPs (Chima 2018a; Montgomery v Lanarkshire Health Board, 2015). Furthermore, according to Beauchamp and Childress (2009), specific requirements should be met, to validate the quality of IC obtained during medical practice. It has been suggested that South African physicians do not meet all requirements of IC, although most doctors substantially met many of the requirements.

Therefore, the authors queried if fulfilment of IC process as expected by law exists (Henley et al. 1995). Reasons for litigations among aesthetics practitioners, and successful award of damages, were reportedly associated with inadequate disclosure of information in respect of IC, in comparison to litigation based on medical negligence (Park et al. 2016). Furthermore, Siddiqui, Shaikh and Memon (2010) documented that quality of IC was invariably less than the expected standards while showing that IC was not obtained from some healthcare users, with recommendations to educate doctors and healthcare users alike.

Chima (2013, 2017, 2018a, 2018b) previously enumerated some challenges faced by doctors and nurses when obtaining IC in South Africa, which included patient workload, language difficulties, lack of administrative support in the form of interpreters and poorly educated patients. Henley et al. (1995) in another study among South African doctors similarly observed that 25% of doctors perceived language as an obstacle to IC. Delany (2005) in a study from Australia enumerated various models of obtaining IC, based on autonomy and a spectrum of reflections and actions. However, she noted that IC among PTs has generally been based on the extent of benefits to be derived from participation in the treatment programme. The various models of obtaining IC from a practical to a reflective spectrum include event, transparency standard, shared decision-making, process and conversation models (Delany 2005). Delany further highlighted that communication at any point in time with the expected treatment outcome might influence the model engaged in the process of obtaining IC during physiotherapy treatment. In light of ethics and practice, Delany et al. (2010) recommended the use of the ‘Active Engagement Model’ to narrow the difference between knowledge of ethics and practice. The NHA together with the HPCSA guidelines stipulate the essentials of full information disclosure during the IC process. Such essentials include information regarding diagnosis, risks and benefits of treatment, cost, options available and risks of refusing treatment, among others (HPCSA 2008; NHA 2003).

Furthermore, section 6(2) of the NHA requires that such information be provided, ‘in a language that the user understands and in a manner which takes into account the user’s level of literacy’ (NHA 2003:s.6(2). Competency of the patient, understanding, appreciation of information shared and voluntariness in decision-making are, however, required preconditions before IC can be considered valid (Beauchamp & Childress 2009; Chima 2018a; Dhai & McQuoid-Mason 2011).

With an 8% reported IC-related misconduct charges against PTs, on record at HPCSA (Hoffmann & Nortjé 2015), one needs to ask whether physiotherapy professionals understand what is expected of them in terms of IC. Are there any specific challenges this category of HCPs encounters during clinical practice? Do PTs have knowledge of the laws and guidelines related to IC in South Africa? Is their practice consistent with international codes and local regulations? Is there importance attached to the practice of IC? These questions and others were designed to be answered by this study. Physiotherapists, however, may have difficulty in obtaining quality IC because of various challenges. It appears to be important to document understanding and practice of IC process among physiotherapy professionals. Therefore, this study aimed to describe the understanding and practice of IC among randomly selected PTs, physiotherapists’ assistants (PTAs) and physiotherapists’ technicians (PTTs), within public health institutions in the Ethekwini Health District, South Africa.

## Methods

This study was a quantitative, cross-sectional, self-reported observational study, conducted using a semi-structured, self-administered questionnaire. The questionnaire was designed to examine and describe the practice and attitudes of PTs, PTAs, PTTs and their understanding and knowledge of IC. Excluded were community service PTs who are generally deployed to under-serviced rural or semi-urban settings. As Ethekwini District is a predominantly urban area, this category of PTs was few in number and not readily available for inclusion in our study. Also excluded were staff with academic roles without direct patient contact and clinical duties.

All public health institutions in the Ethekwini District of KwaZulu-Natal (KZN) with practising PTs, PTAs and PTTs, categorised as central, tertiary, regional, district, specialised (Tuberculosis, Psychiatric and Rehabilitation institutions), and community health centres (CHC) were included in the study. Ethekwini District is a predominantly urban area with a metropolitan city (Durban), and outlying semi-urban townships (Chima 2018a; Statistics South Africa 2016). Ethekwini Health District has the largest number of practising PTs, PTAs and PTTs when compared with other KZN health districts, with a total population of 101 PTs and 28 PTTs and PTAs convenient for access to our study. The total number of PTs was 284 and PTTs and PTAs was 42 in all KZN public health institutions (KwaZulu Department of Health 2016, 2018). The target population was government employed HPCSA registered PTs, PTAs and PTTs. A list of the 17 eligible public institutions was extracted with categorisation of level of service noted. A total of 101 PTs and 28 PTAs and PTTs qualified and practising locally were targeted for inclusion.

The sample population was initially estimated at 110 participants with a target response rate of 50%. Twenty-five and 19 PTAs and PTTs were required as a minimum for our study as recommended by a consulting biostatistician. Convenience sampling was used for easy access to the 17 healthcare institutions as they are strategically situated across the suburbs of Ethekwini metropolitan municipality. One hundred and ten questionnaires were therefore administered across the various healthcare institutions.

### Data collection

The questionnaire used in this study was adapted with few amendments of questions from a validated questionnaire for HCPs, previously deployed for studies among doctors and professional nurses in Ethekwini municipality as previously reported (Chima 2013, 2017, 2018a). The questionnaire was presented in three sections: Section A: Demography; Section B: Practices and Attitudes; and Section C: Generic Knowledge and Practice. Amendments to the questionnaire related to questions that were outside the scope of practice of PTs such as ‘age of consent to termination of pregnancy’. Such questions were eliminated or substituted. Inclusion of therapists’ assistants among the HCPs who are capable of obtaining IC was considered and adopted. Awareness of rehabilitation therapists of available policy and patients’ rights-promoting documents was also considered.

A pilot study was conducted among academic university affiliated PTs to ensure that the questionnaire addressed the questions to be answered by this cohort. Content and face validity of the questionnaire was evaluated, with minor changes made to the questionnaire based on responses from the pilot study. Questionnaires from all respondents were distributed and collected within 2 months (May–June 2018), with reminders and repeated visits to study sites to increase response rate. Respondents used themes capable of describing practices and experiences. Conclusions generated were related to our study variables.

### Data analysis

Data were captured and analysed using Statistical Package for Social Sciences (SPSS) version 25 (IBM 2012).

Participant responses were allocated codes, which were then captured for analysis. Results were presented using descriptive statistics of mean, average, median, mode, standard deviation and percentages. Kruskal–Wallis and Mann–Whitney *U* tests were used to calculate significance levels for independent samples. Spearman’s rho and Pearson’s tests were used to calculate correlations between informed consent aggregate score (ICAS) and summated attitude scores. Cronbach’s alpha was used to calculate reliability of questions used for ICAS. The informed consent aggregate score is designed to measure the knowledge of IC by HCPs using 12 items derived from international ethical codes and guidelines on IC, including elements of information disclosure, comprehension and voluntariness, as previously reported by Chima (2013). The ICAS was based on calculations from affirmative responses for information disclosed such as diagnosis, treatment options, recommended treatments, and risks of refusing recommended treatment, general risks, benefits and questions related to capacity, voluntariness, understanding and agreement (Chima 2013, 2018a).

### Ethical considerations

Our study was reviewed and approved by the University of KwaZulu-Natal Biomedical Research and Ethics Committee (Reference No.: BE551/17), and was also reviewed and approved by KwaZulu-Natal Department of Health Knowledge and Research Ethics Committee (Reference No.: HRKM 531/17). Ethekwini District Health Department and institutional heads approval were also obtained, while IC was obtained from all participants. Consent forms were separated from responses and coded for confidentiality.

## Results

### Demographic characteristics

One hundred and ten questionnaires were distributed to potential participants at the targeted public health institutions. Forty-nine questionnaires were received back, with an overall response rate of 44.5%. However, response rates varied from a low of 6.6% to a high of 100% across institutional physiotherapy departments included in our study. The average age of participants was 38.37 years, 93% were female, and distribution across professional ranks included 43 PTs, three PTTs and three PTAs. Mean age of PTs was 36.05 (± 6.758), while PTAs and PTTs was 55 (± 8.741). The mean years of experience across the physiotherapy discipline was 14.031 (± 7.902). Mean years of experience for PTs was 12.965 (± 6.3280), while it was 21.667 (± 13.559) for PTAs and PTTs combined. The majority of PT respondents are at production level *n* = 22 (54%), while supervisory level was *n* = 19 (46%). Thirty (88.4%) PT respondents indicated general practice, while one (2.3%) PT had a variety of special interest areas. Demographic characteristics of respondents are summarised in Table 1.

**TABLE 1:** Demographic characteristics of participants

Characteristics	*n* = 49	Percentage
Physiotherapist	43	88
Physiotherapists’ technician	3	6
Physiotherapists’ assistant	3	6
Male	3	6.1
Female	46	93.8
Average age (years)	38.37, s.d. (± 9.345)	-
Average years of experience	14.031, s.d. (± 7.902)	-

Note: *n*, is number of respondents; s.d., standard deviation.

### Time spent on information disclosure and informed consent forms

Fifty-six per cent (*n* = 27) respondents reported spending between 5 and 10 min to give information to healthcare users, while *n* = 6 (12.5%) respondents spent less than 5 min and another *n* = 11 (23%) spent between 10 and 20 min. Sixty per cent (*n* = 26) of PTs spent 5–10 min to give information, while *n* = 2 (30.3%) PTAs/PTTs spent 10–20 min. The majority of respondents *n* = 36 (73.4%) indicated that time for information disclosure was sufficient, *n* = 10 (20%) indicated that time spent on giving information to patients was not adequate and *n* = 3 (6.1%) reported ‘Don’t know’. Among the PTs *n* = 32 (74.7%) indicated ‘Yes’ to time sufficiency to obtain IC, while *n* = 8 (18.6%) indicated ‘No’; and among the PTTs/PTAs *n* = 4 (66.7%) indicated ‘Yes’, while *n* = 2 (33.3%) indicated ‘No’.

**FIGURE 1:**
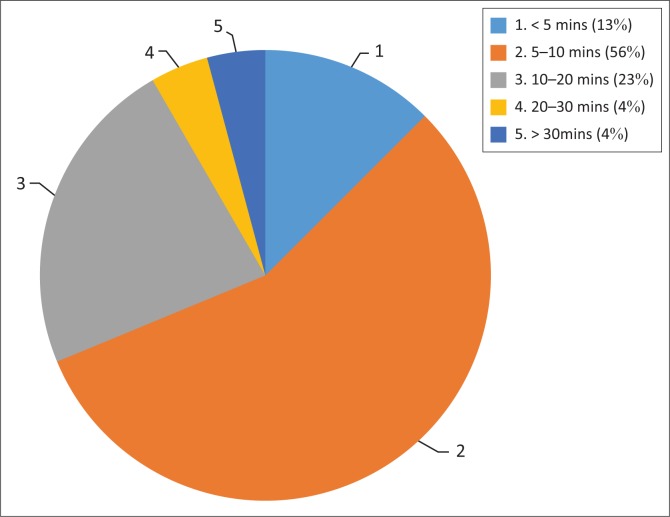
Time spent on information disclosure.

**FIGURE 2:**
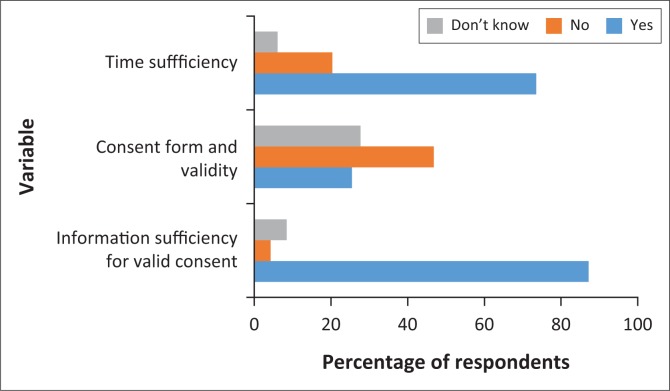
Time sufficiency, information and consent forms used to obtain valid informed consent.

Figure 1 represents time spent on disclosure of information during the IC process. Eighty-seven per cent (*n* = 41) of respondents believed information given was sufficient to obtain valid IC, while *n* = 4 (8.5%) answered ‘Don’t know’ and another *n* = 2 (4.3%) indicated ‘No’. About *n* = 22 (47%) respondents indicated consent forms used were not adequate for procurement of IC, *n* = 13 (27.7%) indicated ‘Don’t know’ and *n* = 12 (25%) of respondents indicated ‘Yes’. Figure 2 represents responses to use of current consent forms to obtain valid IC, sufficiency of time and information disclosed.

### Assessment of competence

**FIGURE 3:**
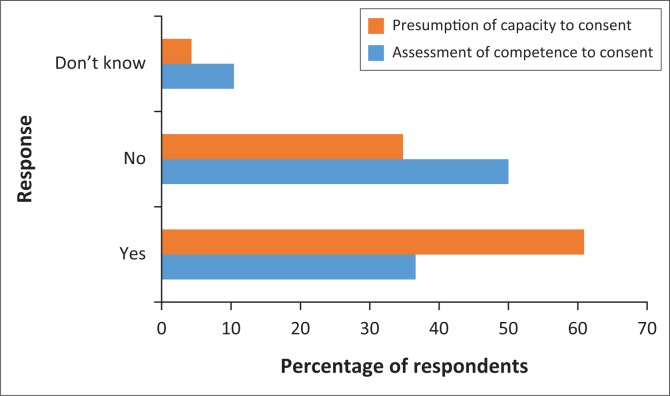
Presumption of capacity to consent and assessment of competence to consent in percentage.

Thirty-six per cent (*n* = 19) of respondents indicated ‘Yes’ to assessment of competence of their patients to consent to treatment, while *n* = 24 (50%) responded ‘No’. Sixty per cent (*n* = 28) indicated ‘Yes’ to presumption of capacity to consent, while *n* = 16 (35%) indicated ‘No’. Figure 3 represents the responses in percentage to presumption of capacity to consent and assessment of competence to consent by physiotherapy professionals.

### Knowledge of age of consent to medical treatment

**FIGURE 4:**
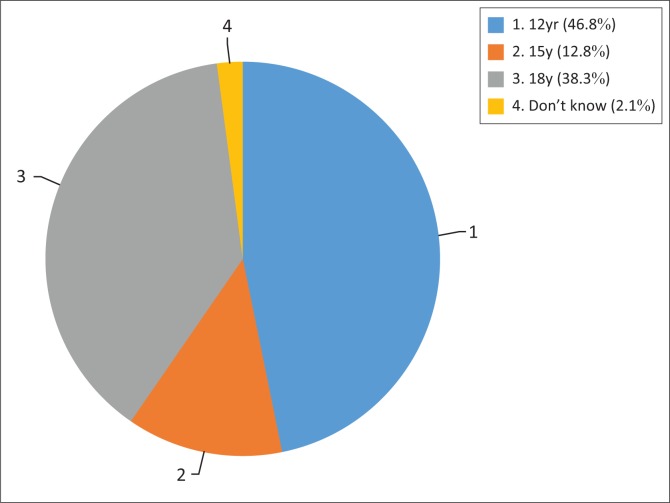
Knowledge of ‘age of consent’ to routine medical treatment.

Forty-seven per cent (*n* = 22) of respondents indicated correct age to consent to medical treatment as ‘12 years’, while *n* = 24 (53%) were incorrect and *n* = 1 (2.1%) responded ‘Don’t know’. Figure 4 represents physiotherapy professionals’ knowledge of ‘age of consent’ to routine medical treatment in South Africa.

### Methods of obtaining informed consent

Methods of obtaining IC were categorised as verbally, written or both. Eighty-nine per cent (*n* = 41) respondents reported using verbal method, *n* = 2 (4.3%) indicated obtaining IC in writing and another *n* = 2 (4.3%) respondents reported obtaining IC using both methods. Another *n* = 1 (2.2%) indicated, ‘It depends’.

### Explanation of benefits, risks and allowing choice of treatment

One hundred per cent (*n* = 49) of respondents explained benefits of treatments to patients, while *n* = 40 (81%) explained common risks. Twenty-seven per cent (*n* = 13) of respondents allowed patients to make a choice of procedure or treatment, *n* = 33 (68.8%) responded ‘No’ to the question: ‘Do you allow your patients to choose a procedure or particular treatment’ and 4% responded ‘Don’t know’. Seventy per cent of respondents explained the most serious risks, while only 31% explained ‘all material risks’.

### Challenges to obtaining informed consent

**FIGURE 5:**
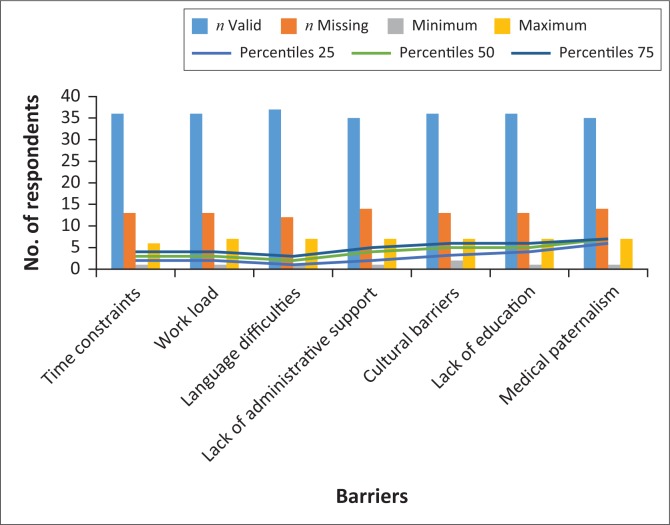
Barriers to obtaining informed consent as reported by respondents.

The challenges to obtaining IC were arranged in a spectrum of high to low, from language difficulties, time constraints and workload, lack of administrative support, cultural barriers, lack of education and finally medical paternalism. The scoring of these challenges was from 1 to 7, where 1 was the highest score and 7 the lowest score. Barriers to obtaining IC as reported by participants are shown in Figure 5.

### Obtaining informed consent in emergency cases

Twenty-seven per cent (*n* = 12) participants responded ‘Yes’ when asked, ‘Do you obtain IC in emergency cases?’ Twenty-nine per cent (*n* = 13) indicated ‘No’, while *n* = 9 (20%) and *n* = 11 (24%) indicated ‘Don’t know’ and ‘It depends’, respectively.

### Informed consent aggregate scores

**FIGURE 6:**
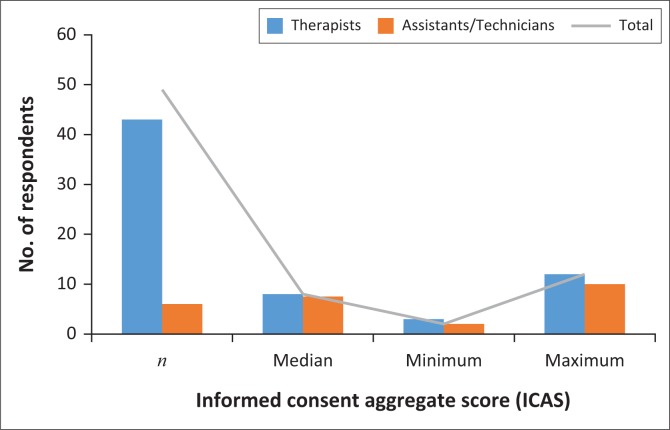
Informed consent aggregate score distribution between physiotherapists, physiotherapists’ technicians and physiotherapists’ assistants.

Informed consent aggregate scores ranged between a minimum of two to a maximum of 12. The distribution of ICAS between the PTs, PTTs and PTAs was the same, hence there is no significant difference *ρ* = 0.19 at a significance level of *ρ* < 0.05, 95% confidence interval. ICAS scores between PTs, PTTs and PTAs is shown in Figure 6. Comparison of ICAS with the time spent on information disclosure showed no significant difference between ICAS and time spent on information disclosure across the PTs, PTTs and PTAs categories, *ρ* = 0.35 with significance level at *ρ* < 0.05 (Figure 6).

### Attitude to informed consent by physiotherapists

The summated attitude score was used to calculate the attitude of physiotherapy professionals to IC and presented as values, where lower calculated scores signified a satisfactory attitude, and higher scores denote an unsatisfactory attitude. Physiotherapists’ attitude was scored according to the definite categorical response of ‘Yes’ and ‘No’, and non-definite categorical response of ‘I don’t’ know and ‘I don’t think so’. These responses were based on five selected questions relating to giving adequate explanations to patients, patients’ preference of treatment options, patients’ understanding of information given, competence of patients to consent and presumption of capacity to consent, by physiotherapy professionals.

### Correlation between informed consent aggregate scores and summated attitude score

Summated attitude scores were derived from questions related to information given to patients, such as ‘Do you think information provided is sufficient to provide valid IC?’; choice of treatment by patients (‘Do you allow your patients to choose a procedure or particular treatment?’); understanding (‘Do you think patients understand information given to them?’); assessment of competence (‘Do you routinely assess competence of patient to consent to treatments?’); and presumption of capacity to consent (‘Do you generally presume patients have the capacity to consent to medical treatment?’). Responses were directly compared with ICAS score among PTs, PTTs and PTAs. Table 2 presents correlation between ICAS and summated attitude scores among participating physiotherapy professionals.

**TABLE 2:** Correlation between informed consent aggregate score and summated attitude score.

ICAS correlation	Adequate explanation	Choice of treatment by patients	Understanding by patients	Competence assessment	Presumption of capacity
Pearson correlation PTs	0.277	0.344*	0.639**	0.140	0.392**
Pearson correlation PTAs and PTTs	0.000	0.548	0.659	0.536	0.866*
Non parametric Spearman’s rho PTs	0.284	0.266	0.659**	0.090	0.407**
Non parametric Spearman’s rho PTAs and PTTs	0.131	0.621	−0.289	0.775	0.655

ICAS, informed consent aggregate score; PTs, physiotherapists; PTAs, physiotherapists’ assistants; PTTs, physiotherapists’ technicians.

* , Correlation significant at 0.05 level (2-tailed).

** , Correlation significant at 0.01 level (2-tailed).

### Relevance, language used and responsibility to obtain informed consent

The relevance of IC among the respondents was scored at a median of 5, while minimum and maximum scores were 1 and 5, respectively. Seventy-seven per cent (*n* = 38) respondents indicated healthcare provider and patients do have a joint responsibility to obtain IC, and another *n* = 38 (77%) respondents used both English and a local language to obtain IC.

### Recommendations and suggestions indicated by respondents on informed consent

One open-ended question included in the questionnaire asked participants: ‘Do you have any suggestions or recommendations regarding IC?’ Suggestions and recommendations respondents gave included the following:

- ‘Do more in-service on importance of IC + (and) implement it’
- ‘Ask the patient for consent’
- ‘Have a section @ (at) the top of the assessment form to indicate if consent was asked by the PT and given by the patient’
- ‘To have written forms to be signed’
- ‘It (IC) should be included during the initial assessment & (and) should be written in the patient’s language’
- ‘Essential part of preparation of patient interview prior to starting rehabilitation’
- ‘Must be obtained prior to consultation, that is, when pt. (patient) is in waiting area; are they willing to go thru (through) with Rx (treatment) and if they are unaware of Rx – it should be explained’
- ‘General consent form easily available & (and) given to patients when they attend registration desk in both English and isiZulu (local language) in separate copies’

## Discussion

Informed consent is an ethical and legal obligation requiring competence, adequate explanation, understanding, appreciation and voluntary acceptance of information shared and intervention proposed before medical treatment (Dhai & McQuoid-Mason 2011). Cases leading to litigation in healthcare practice previously were related to death, serious injuries and harm, but in recent times, expectations of patients with a measure of dissatisfaction coupled with awareness of rights have prompted more claims based on negligence (Chima 2011; Hoffmann & Nortjé 2015; Park et al. 2016). Hence, IC remains a necessary contract, to be fulfilled at all times between PTs or other HCPs and patients (WHO 1999). This contract, informed by knowledge of the process, requires that valid IC of an acceptable quality should be obtained before medical treatment.

Informed consent can create a contract enforceable by law if consent agreements contain provisions where ‘mutual assent, offer, acceptance, and consideration exist’ (Chima 2006, 2018a; Grimes v Kennedy Krieger Institute Inc. 2001). Disputes emanating from inadequate IC with dissatisfaction of healthcare users can result in litigations for employers and employees. Therefore, healthcare employers have a role to play in ensuring that knowledge and understanding of legislation and guidelines essential to ethical and legal practice are demonstrated by their employees (Park et al. 2016). However, professionalism requires that PTs, PTTs and PTAs conform to expected administrative standards, with barriers to obtaining IC considered and effectively eliminated (Agu et al. 2014). To achieve treatment goals successfully, prevention of foreseeable harm and risks and ensuring autonomy for healthcare users through IC is necessary (Cordasco 2013). This empirical study on IC conducted among PTs and therapy assistants has been able to evaluate the practice of some government employed PTs and therapist assistants within the Ethekwini District, KZN, in relation to time spent, knowledge, understanding, availability and use of policies, and documentation instruments. The implications of this relate to professional practice in accordance with expected norms and promotion of such. However, our study also showed that therapist assistants who participated, and by scope of practice are supervised by PTs during rehabilitation and monitoring of patients, also obtain IC from patients as expected, according to applicable legislation and guidelines.

Among the physiotherapy professionals who participated in this study, time spent to obtain IC varied, with the majority spending between 5 and 10 min. Almost 75% of the respondents indicated time spent on IC was enough. The reasons stipulated for inadequate time for IC included high numbers of patients to be attended to, acute phase of patients’ conditions, which affected comprehension, and limited time for disclosure of vital and necessary information. Other factors were patients’ comprehension of information disclosed and language barriers. The challenges of inadequate time were a disadvantage to the quality and practice of IC. While information disclosure may increase patient satisfaction, inadequacy of time may also negatively influence patient satisfaction. Furthermore, there was no way to differentiate between the exact lengths of time spent by physiotherapy professionals on IC, as opposed to the entire clinical encounter. However, in a study among doctors in the United States, the time for an average clinical encounter in general practice was reported as 15 min (Chima 2018a; Kaplan 2004), while the average time for a typical clinical encounter between HCPs and healthcare users was estimated at about 14 min, based on the KZN Department of Health Strategic Plan (Chima 2018a; KZN Department of Health 2010). Previous studies by Chima (2013) and Henley et al. (1995) conducted among medical doctors indicated time spent to obtain IC among the majority of respondents was also between 5 and 10 min.

The model of IC used is critical to optimising the time available to obtain valid IC. Models such as the Event and Shared decision-making can substitute for inadequate time. These two models, according to Delany (2005), are practical and targeted with values in the form of communication sets used, such as the use of generic forms in the Event model, and reduced information and power asymmetry with increased sense of autonomy and control over treatment decisions in the Shared decision-making model.

The majority of respondents indicated that IC was obtained verbally from patients. About half of the respondents indicated that consent forms are either not available or are not being used in their departments or treatment units. Informed consent aggregate score among PTs, PTTs and PTAs showed no significant difference. This may indicate that information given to patients is the same among all categories of physiotherapy professionals. The information required to be given to patients, which included diagnosis, treatment goals and options, recommended treatments, risk of refusing treatments, risks, recommended treatments, benefits, rights of refusal and costs, were based on the ethical guidelines (HPCSA 2008) and requirements of the NHA (2003).

The majority of physiotherapy professionals provided information on treatment benefits and risks. This may further demonstrate the importance of the values of beneficence as the basis for IC among physiotherapy professionals. There is, however, an indication that not allowing patients to choose procedures, or make a choice in their own treatment, based on the availability of options, may be denying the patient the right to autonomy.

Most of the respondents in this study did not allow patients to make a choice of treatment or procedure.

However, PTs may have a better understanding of what intervention is best for their patients, especially where there may be a lack of many options for treatment, as prevalent in resource-constrained settings like South Africa.

There was no significant difference in the distribution of ICAS between PTs, PTTs and PTAs. This is similar to the ICAS scores observed between professional and enrolled nurses, as previously reported (Chima 2017).

However, ICAS does not fully demonstrate the differing roles different categories of HCPs have during the process of information disclosure and the types of information disclosed. Nonetheless, PTs, PTTs and PTAs are capable of providing the necessary information needed to procure valid IC.

Informed consent being a doctrinal requirement among all HCPs, and entrenched in the legislation, may not be ignored, as this is a contract between healthcare providers and healthcare users. However, information disclosed during IC and the barriers to IC, which include language, workload and time constraints, should be addressed to improve on the practice of IC, and avoid potential lawsuits that may arise from inadequate information disclosure. Park et al. (2016) documented the necessity of having adequate disclosure of information as a possible remedy to the awarding of damages against offending HCPs. However, the majority of PTs in this study indicated they obtained IC verbally, even though the practice of physiotherapy may involve substantial risks. Given the increased awareness of patients around human rights and litigation within the healthcare sector, disclosure of the benefits of treatment alone should not constitute the major aspect of IC among physiotherapy professionals. Moreover, documentation, which reflects that IC has been obtained, should be a matter of necessity because documented IC processes have the capability to withstand the rigor of legal interrogation.

Challenges to obtaining IC include workload, language barriers and inadequate time. These challenges illustrate the demand for healthcare services, the multilingual and complex multicultural society that is South Africa (Chima 2018a, 2018b; Schlemmer & Mash 2006) and the demand on PTs’ time. In the absence of adequate administrative support such as trained interpreters, a need to spend time on non-clinical-related issues will arise, thereby resulting in increased workload and decreasing available time for clinically related tasks (Chima 2017, 2018a, 2018b). Language difficulty had a high ranking in the barriers to IC, followed by workload. In previous South African studies by Henley et al. (1995), only 25% of doctors perceived language as a barrier, while in another study by Chima (2013), 88% indicated language as a barrier and 82% considered lack of interpreters as another barrier to obtaining IC by doctors. The multicultural and metropolitan setting of Ethekwini District and improving access to healthcare institutions may be indicative of the language barriers experienced. The majority of the respondents in our study felt it is the responsibility of the healthcare providers generally to obtain IC, while 25% of respondents felt it is the responsibility of the patient.

In a similar study by Henley et al. (1995), the majority of doctors (*n* = 795) felt it was their responsibility to obtain IC. These differing views on whose responsibility it is to obtain consent may influence the practice of IC among PTs, PTTs and PTAs at public health institutions in KZN Province and other similar settings in South Africa.

The legal age of consent to routine medical treatment according to current South Africa law is 12 years (Children’s Act 2005). More than half of the population of PTs and assistants who participated in our study (53%) do not have such knowledge. In a similar study among professional nurses in Ethekwini, only about 25% of nurses correctly identified the legal age of consent to treatment in South Africa (Chima 2017). This inadequate knowledge of the age of consent to medical treatment may greatly undermine the practice of IC, as it may result in the violation of the rights of the healthcare users, especially children, because of lack of knowledge regarding the provisions of current legislation. This lowered age of consent was designed by government to take into consideration the issue of child-headed households in South Africa arising consequent to the HIV/AIDs pandemic (Brand South Africa 2007; Chima 2018a). It may be presumed, however, that PTs and PTTs/PTAs who indicated the incorrect age of 18 years of age as consent to medical treatment assume that the age of legal capacity is the same as the age of majority in South Africa (Brand South Africa 2007).

Moreover, the majority of respondents do not assess the competence of their patients. About one-third presume the patients have given prior IC, before being referred to PTs for care.

Physiotherapists, PTAs and PTTs tend to explain benefits of treatment during IC and therefore lean towards beneficence and non-maleficence (Delany 2005), rather than respect for autonomy (Chima 2009), as a basis for ethical practice. A high number of respondents indicated that IC is relevant to the practice of physiotherapy in this setting. All respondents gave an indication that benefits of the treatment were disclosed to the patients.

Normal physiotherapy practice requires adherence of patients to treatment programmes and explanation of benefit may assist to get maximum cooperation from patients. Cooperation of the patient may not be limited to satisfaction, but may also assist in achieving intervention goals (Delany 2005).

The explanation of benefits of treatments by PTs can facilitate positive outcomes, and may be responsible for most PTs highlighting the benefits of treatments during the IC process. This may indicate that self-determination was not the primary reason why IC is obtained, but is done for the benefit of getting patients’ participation and adherence, and may impact the treatment outcomes. Verbal methods of obtaining IC were high among this cohort of PTs and assistants with the majority obtaining verbal consent without documentation. While it is acceptable to obtain IC verbally, it may be essential to consider the risks associated with a particular clinical intervention, to determine the most appropriate method of documenting IC. Litigious cases adjudicated, as reported by Park et al. (2016), are often based on a test of complete disclosure of information, and not necessarily on malpractice. Hence, poor documentation of IC during clinical practice may not be able to withstand rigorous cross-examination on litigation in a court of law.

Dehghan et al. (2013) reported the impact of the installation of a ‘Patients’ Rights Charter’ in areas where patients receive services to enhance promotion of patients’ rights towards quality healthcare. Identified factors to improve the promotion of patients’ rights in their study included ethics courses, workshops and in-service training to facilitate patients’ knowledge of their rights. It was, however, determined that no statistically significant relationship existed between educational level, areas of practice and work experience among occupational therapists and their knowledge of patients’ rights. Multilateral approaches to ethical care can, therefore, be encouraged by the promotion of patients’ rights. Rights to information, privacy and responsibilities are included in the Patients’ Rights Charter of South Africa (Chima 2018a; KZN Department of Health 2017).

Sixty-four per cent of participants in a study by Lledo et al. (1998) reportedly fulfilled patients’ rights, while only 84% were aware of such patients’ rights. Khoza (2009) reported in another study among hospital patients in Gauteng Province that 37% indicated that Batho Pele principles (South Africa Department of Public Service and Administration 1995) were clearly displayed; 35% were not sure, and 28% indicated Batho Pele principles were not clearly displayed. The awareness of availability of documents, such as Batho Pele principles, was lacking among 50% of the participants in our study. Questions designed to evaluate attitude of PTs could be aligned to measure PTs’ attitude to awareness of the work environment, basic understanding of tools to promote patient education and satisfaction of criteria for valid IC. It is critical that patients have a satisfactory understanding of explanations given to them by their PTs for IC to be consistent with local regulations and international codes. Physiotherapists and assistants in this study showed a relatively satisfactory attitude as the summated attitude scores obtained were generally low, which signified positive attitudes towards IC.

## Limitations of this study

A minimum number of 25 respondents were initially estimated for each category of PT, PTTs and PTAs. However, low response rates for the PTAs and PTTs was observed because of job attrition at study sites or lack of interest to participate in our study. The number of respondents may have influenced the importance and interpretation of the results. A larger study with an increased population of physiotherapy professionals could be conducted in future to validate results obtained from this study. Furthermore, studies among PTs, PTTs and PTAs working in the private healthcare sector or in more rural settings may provide a different perspective on the practice of IC among PTs and their assistants. Nevertheless, the results of this empirical study provide a current snapshot of the understanding and practice of IC among PTs and their assistants at public health institutions in KZN Province, South Africa.

## Conclusions

The majority of respondents in this study indicated that time spent on information disclosure and IC was 5–10 min.

Moreover, the majority considered this time adequate. Most respondents were also of the opinion that IC is relevant to physiotherapy practice. The significance of relevance may highlight attitudes towards IC. The perception that patients’ outcome during physiotherapy intervention should be based on participation and information sharing is crucial to patients’ engagement, adherence and improved patient outcomes.

Informed consent is part of international clinical best practice, entrenched in local laws, regulations and guidelines. However, a high number of participants in this study were unaware of the availability of policy documents that display information regarding patients’ rights within their working environment. Unawareness of such displays within a service area may be an indication of lack of interest in clinical governance tools.

Physiotherapy professionals demonstrated insufficient knowledge of IC in accordance with the provisions of current legislation. Barriers to IC identified in this cohort include language and excessive workload. It is therefore recommended that regular updates on ethics and healthcare law can be an intervention to improve this knowledge gap. Continuing professional education on IC with adoption of an ethics training model, such as active engagement, may be crucial to improve knowledge and practice of IC by physiotherapy professionals in the resource-constrained settings prevalent in African and other developing countries.
